# How automatic activation of emotion regulation influences experiencing negative emotions

**DOI:** 10.3389/fpsyg.2015.01628

**Published:** 2015-10-27

**Authors:** Dorota Kobylińska, Dorota Karwowska

**Affiliations:** Faculty of Psychology, University of WarsawWarsaw, Poland

**Keywords:** automatic emotion regulation, emotion control, negative emotion, positive emotion, emotion expression

The existence of automatic, unconscious processes influencing human emotion, cognition, and behavior is widely accepted and confirmed by numerous studies (e.g., Ohme et al., 2001; Jarymowicz, 2002; Hassin et al., 2005; Wentura and Degner, 2010; Bargh et al., 2012; Bargh, 2014; Wentura and Rothermund, 2014). Researchers demonstrated the mechanisms of implicit social cognition, including implicit attitudes and stereotypes (e.g., Greenwald and Banaji, 1995; Nosek et al., 2009; Molden, 2014), implicit affect (e.g., Zajonc, 1980, 2004; Murphy and Zajonc, 1993; Feldman-Barrett et al., 2005; Ohme, 2005; Kobylińska, 2007; Kolańczyk, 2007), as well as automatic processes in perception, evaluation and motivation (Bargh, 1997; Bargh et al., 2012; Rotteveel et al., 2015). Such mechanisms are described as automatically occurring and hard to control or modify. However, more and more researchers (Baumeister and Vohs, 2004; Koole, 2009; Kobylińska and Karwowska, 2014; Jarymowicz and Imbir, 2015) are starting to explore whether an individual always remains under the unconscious, automatic influence of these processes. Even if intentional, voluntary control is not possible, some evidence suggests that automatic self-regulation, including emotion regulation (which does not require person's conscious knowledge and intention, Fitzimons and Bargh, 2004; Koole and Rothermund, 2011) can operate outside of conscious awareness and influence the course of other processes (e.g., emotion) even the implicit ones (e.g., Kolańczyk and Pawłowska-Fusiara, 2002; Kobylińska, 2007; Wentura and Degner, 2010; Teige-Mocigemba and Klauer, 2013; Koole et al., 2015).

In this opinion article we base our understanding of emotion regulation on the process model of emotion regulation proposed by Gross (2008, 2014, 2015a,b), who defined emotion regulation as a series of processes which cause changes in the dynamics, duration and speed of emotional activation, as well as changes in accompanying behavior, experience, and physiology. Accordingly, Gross (2008) argued that regulation could result in decreasing, intensifying or sustaining of either positive or negative emotions. He distinguished between antecedent-focused and response-focused regulation showing evidence that the antecedent-focused strategies (e.g., reappraisal) are more effective and adaptive then the response focused strategies (e.g., suppression) (Gross et al., 2006). More and more researchers, including ourselves, agree that emotion regulation manifests itself in a flexibility in experiencing positive and negative emotions by using different emotion regulation strategies (e.g., Ekman and Davidson, 1994; Bonanno and Burton, 2013; Sheppes et al., 2014; Aldao et al., 2015). Still, the majority of current research concentrates on the regulation of negative emotions, as decreasing the intensity or duration of negative emotions seems especially important for people in a present world (Gross, 2015a). We also focus on regulation of negative emotion. Our aim is to present some results that indirectly suggested the existence of automatic emotion regulation as well as some direct evidence (including replication) for this kind of regulation. Results of our own experiments described below add to the discussion about automatic emotion regulation showing consensus with previous studies.

## Automatic emotion regulation—indirect evidence

In recent years, there has been an increasing interest in research on self-regulation in general (Baumeister and Vohs, 2004; Gross, 2015a). Some of the researchers began to report that self-regulation may in fact also happen on the automatic or implicit level (Fitzimons and Bargh, 2004; Koole and Coenen, 2007). Studies presented by Bargh and colleagues (Fitzimons and Bargh, 2004; Gollwitzer and Bargh, 2005) showed that the so called higher psychological processes, such as achieving goals or following norms and values, can be activated in response to a triggering event, without the individual's awareness and intention and influence his or her perception, emotion, thinking, and behavior (Bargh, 1997, 2014; Bargh et al., 2001; Aarts and Dijksterhuis, 2003). They described the auto-motive model, according to which goals are represented in the mind and can be automatically activated (for example, by priming), influencing the person's behavior outside of their conscious awareness. Research on automatic activation of goals and norms is an important basis for the hypothesis about the existence of automatic regulation of emotions (Fitzimons and Bargh, 2004; Bargh and Williams, 2007). It suggests that when goals or norms referring to regulating emotions are formed in an individual's mind, they can be activated without his or her awareness and influence an emotional response (see also: Mauss and Tamir, 2014). However, although studies described above give a broader context for understanding how automatic emotion regulation may work, they do not directly prove its existence.

Mauss et al. (2007, 2008) defined automatic regulation of emotions as a goal-activated change of any aspect of emotion without a conscious decision, without directing attention to the process of regulation and without the engagement of deliberate control. It usually occurs due to automatic activation of the goal of changing the emotional process (Williams et al., 2009). Gyurak et al. (2011) propose a dual-process framework for emotion regulation, arguing that it can be observed both at explicit and implicit level. They claim that the automatic emotion regulation, similarly to the conscious regulation, can involve changes on different stages of the emotional process, including attention deployment, cognitive interpretation, or modulation of emotional reaction and can effectively change the experiential, psychophysiological, or behavioral aspects of the emotion.

In quite a few papers the indirect evidence for automatic emotion regulation was described (Fitzimons and Bargh, 2004; Koole and Jostmann, 2004; Koole and Coenen, 2007; Karwowska and Kobylińska, 2014; Kobylińska and Karwowska, 2014). Fitzimons and Bargh (2004) argued that some of the earlier studies, for example showing the increase in self-esteem under conditions of threat to one's group, could in fact be interpreted using the framework of automatic regulation of emotions. Some researchers explain the effects of the declined influence of affective stimuli on judgments, in people with certain traits or in certain situational contexts, with the concept of automatic regulation (Koole and Jostmann, 2004; Koole and Coenen, 2007; Karwowska and Kobylińska, 2014; Kobylińska and Karwowska, 2014). For example, Koole and Coenen (2007) show that action-oriented individuals (Kuhl, 1992) are good at spontaneous regulation of negative affect (by reducing its intensity when it is required). According to the authors emotion regulation is a learned, habitual skill in action-oriented individuals, applied with no conscious awareness of doing it. Another study, in which action orientation was found to facilitate a decrease of the influence of affect induced through subliminal priming (Jostmann et al., 2005), supports the notion that the regulation associated with the trait of action orientation may be automatic (as action oriented participants were not aware they were regulating their affect but still they were less influenced by it). Such indirect evidence for the existence of automatic regulation comes also from our research. In three experiments (Kobylińska, 2007), participants whose self-control standards were activated before they performed affective priming task were less susceptible to implicit affective stimuli (faces expressing disgust or joy presented for 16 ms) than participants from the control group. Importantly, participants were not aware of the influence of the manipulation of self-control standards on the subsequent task. Other studies showed that effects of suboptimal priming was be reduced by the activation of the reflective system of evaluation (Jarymowicz and Kobyliñska, 2005; Karwowska and Kobylińska, 2014) or in people with certain personality characteristics, such as a high level of cognitive self-distinctiveness, (Karwowska, 2007) or a high level of using reappraisal habitually (Kobylińska and Dyderska, 2009). We interpreted the findings using the concept of automatic emotion regulation.

## Automatic emotion regulation—direct evidence

The results of research described above are problematic to be generalized, since automatic regulation of emotions was not measured, but merely inferred. Soon, other researchers started to more directly address the topic of automatic emotion regulation, showing more evidence for its existence. Mauss et al. (2006) conducted a study based on the assumption of a relationship between the automatic goal of emotion regulation and the implicit attitude toward regulation. They determined the strength of the implicit positive attitude (applying a version of Implicit Association Test), and measured the consequences of anger induced through experimental manipulation. Both the experience of anger and cardiovascular reaction were weaker in individuals with a stronger implicit positive attitude toward emotion regulation, which the researchers explained by the mechanism of automatic regulation of emotions. In another study (Mauss et al., 2007) the goals of either control or expression of emotions were primed (participants were unaware of the manipulation they were subjected to), using a scrambled sentences task (procedure based on Bargh et al., 2001). In the task participants have to form sentences out of given words. In majority of the sentences words are associated with a concept that is to be primed (in this case either emotion control or emotion expression). The results showed that the automatic activation of emotion control resulted in lower level of experienced anger and weaker cardiovascular response than emotion expression priming, which we consider as a strong evidence for the effectiveness of automatic emotion regulation.

As studies using priming in social psychology are discussed more and more often and not all the researchers managed to replicate, for example, the influence of priming on motivation or behavior showed by Bargh (Yong, 2012; Rotteveel et al., 2015), we decided to replicate the effect of primed emotion control on experienced emotions (Kobylińska and Duszyñska, 2013). Based on the studies described above we predicted that automatic activation of emotion control in a negative situation would enable a reduction in negative emotions and an increase in positive emotions. At the beginning of the experiment either emotion control or emotion expression was primed (each in a separate experimental group) by the scrambled sentences procedure (based on Mauss et al., 2007). Participants were not aware that the first part of the study was connected to a second one. In the second part anger was induced by a frustrating task in which achieving success was very difficult. Emotions were measured by the Positive and Negative Affect Scale (Watson et al., 1988) at the beginning and at the end of experiment. While there were no significant differences in the level of experienced negative emotions at the beginning of the study, it was found that after the task aimed at inducing anger individuals from emotion expression group declared a higher level of negative emotions than did individuals from emotion control group. As for positive emotions, there was no significant difference between the two groups after the task. Nonetheless, in the emotion expression group there was a reduction in the level of experienced positive emotions after the task compared to the starting level. This effect did not occur in the emotion control group. It is possible that the lack of differences in positive emotions in emotion control group indicates the effectiveness of the automatic activation of control in preventing from the decrease in positive emotions. Thus the results suggest that the activation of emotion control allows for coping with the increase of negative emotions and with the decrease of positive emotions in the negative situation, which is in line with previous study by Mauss et al. (2007) and proves the existence and the effectiveness of automatic emotion regulation. This conclusion still requires further research.

## Future directions

The research procedure applied in the study by Mauss and in our study does not allow for the determination of which type of emotion regulation (response-focused or antecedent focused) or which emotion regulation strategy (e.g., reappraisal or distraction) was activated by the priming task, and consequently, which type of automatic regulation is effective (Sheppes and Gross, 2012). Mauss et al. (2008) propose, for example, introduction of automatic activation of emotion strategies from different levels of the Gross's model, e.g., reappraisal (from the level of cognitive change), described as an adaptive strategy and a method of effective emotion control, or suppression (from the level of reaction modification), described as a rather maladaptive strategy, related to some negative social, emotional, and cognitive consequences (Williams et al., 2009). Recent research by Chrisou-Champi et al. (2015) show that using reappraisal can be trained and used later spontaneously, which suggests that this strategy can become automatized. Experiments in which the specific strategies of emotion regulation are automatically activated are of our further interest and are being conducted in our lab.

## Funding

Article was prepared thanks to funds from BST 174408-2015 Faculty of Psychology, University of Warsaw.

### Conflict of interest statement

The authors declare that the research was conducted in the absence of any commercial or financial relationships that could be construed as a potential conflict of interest.

## References

[B1] Aarts H. Dijksterhuis A. (2003). The silence of the library: environment, situational norm, and social behavior. J. Pers. Soc. Psychol. 84, 18–28. 10.1037/0022-3514.84.1.18 12518968

[B2] Aldao A. Sheppes G. Gross J. J. (2015). Emotion regulation flexibility. Cogn. Ther. Res. 39, 263–278. 10.1007/s10608-014-9662-4

[B3] Bargh J. (1997). The automaticity of everyday life, in The Automaticity of Everyday Life: Advances in Social Cognition, Vol. 10, ed Wyer R. S. Jr. (Mahwah, NJ: Erlbaum), 1–61.

[B4] Bargh J. A. (2014). Our unconscious mind. Sci. Am. 30, 30–37. 10.1038/scientificamerican0114-30 24616968

[B5] Bargh J. A. Gollwitzer P. M. Lee-Chai A. Barndollar K. Trötschel R. (2001). The automated will: nonconscious activation and pursuit of behavioral goals. J. Pers. Soc. Psychol. 81, 1014–1027. 10.1037/0022-3514.81.6.1014 11761304 PMC3005626

[B6] Bargh J. A. Schwader K. L. Hailey S. E. Dyer R. L. Boothby E. J. (2012). Automaticity in social-cognitive processes. Trends Cogn. Sci. 16, 593–605. 10.1016/j.tics.2012.10.002 23127330

[B7] Bargh J. A. Williams L. E. (2007). Nonconscious regulation of emotion, in Handbook of Emotion Regulation, ed Gross J. (New York, NY: The Guilford Press), 429–445.

[B8] Baumeister R. F. Vohs K. D. (eds.). (2004). Handbook of Self-Regulation. New York, NY: The Guilford Press.

[B9] Bonanno G. A. Burton C. L. (2013). Regulatory flexibility: an individual differences perspective on coping and emotion regulation. Psychol. Sci. 8, 591–612. 10.1177/1745691613504116 26173226

[B10] Chrisou-Champi S. Farrow T. F. Webb T. L. (2015). Automatic control of negative emotions: evidence that structured practice increases the efficiency of emotion regulation. Cogn. Emot. 29, 319–331. 10.1080/02699931.2014.901213 24678930 PMC4241596

[B11] Ekman P. Davidson R. J. (eds.). (1994). The Nature of Emotion: Fundamental Questions. New York, NY: Oxford University Press.

[B12] Feldman-Barrett L. Niedenthal P. M. Winkielman P. (2005). Emotion and Consciousness. New York, NY: The Guilford Press.

[B13] Fitzimons G. M. Bargh J. A. (2004). Automatic self-regulation, in Handbook of Self-Regulation, eds Baumeister R. F. Vohs K. D. (New York, NY: The Guilford Press), 151–170.

[B14] Gollwitzer P. M. Bargh J. A. (2005). Automaticity in goal pursuit, in Handbook of Competence and Motivation, eds Elliot A. Dweck C. (New York, NY: Guilford Press), 624–646.

[B15] Greenwald A. G. Banaji M. R. (1995). Implicit social cognition: attitudes, self-esteem and stereotypes. Psychol. Rev. 102, 4–27. 10.1037/0033-295X.102.1.4 7878162

[B16] Gross J. J. (2008). Emotion regulation, in Handbook of Emotions, 3rd Edn. eds Lewis M. Haviland-Jones J. M. Feldman-Barett L. (New York, NY: Guilford Press), 497–512.

[B17] Gross J. J. (ed.). (2014). Handbook of Emotion Regulation, 2nd Edn. New York, NY: Guilford Press.

[B18] Gross J. J. (2015a). Emotion regulation: current status and future prospects. Psychol. Inq. 26, 1–26. 10.1080/1047840X.2014.940781

[B19] Gross J. J. (2015b). The extended process model of emotion regulation: elaborations, applications, and future directions. Psychol. Inq. 26, 130–137. 10.1080/1047840X.2015.989751

[B20] Gross J. J. Richards J. M. John O. P. (2006). Emotion Regulation in everyday life, in Emotion Regulation in Couples and Families: Pathways to Dysfunction and Health, eds Snyder D. K. Simpson J. Hughes J. N. (Washington, DC: American Psychological Association), 13–35.

[B21] Gyurak A. Gross J. J. Etkin A. (2011). Explicit and implicit emotion regulation: a dual-process framework. Cogn. Emot. 25, 400–412. 10.1080/02699931.2010.544160 21432682 PMC3280343

[B22] Hassin R. R. Uleman J. S. Bargh J. A. (eds.). (2005). The New Unconscious. New York, NY: Oxford University Press.

[B23] Jarymowicz M. (2002). On the benefits from research on implicit affective information processing. Pol. Psychol. Bull. 33, 5–11.

[B24] Jarymowicz M. Imbir K. (2015). Toward a human emotions taxonomy (based on their automatic vs. reflective origin). Emot. Rev. 7, 183–188. 10.1177/1754073914555923

[B25] Jarymowicz M. Kobylińska D. (2005). Z badań nad wpływem utajonego afektu na formułowane sądy w warunkach uprzedniego pobudzania do refleksyjności. Stud. Psychol. 43, 25–40.

[B26] Jostmann N. B. Koole S. L. van der Wulp N. Fockenberg D. A. (2005). Subliminal affect regulation: the moderating role of action versus state orientation. Eur. Psychol. 10, 209–217. 10.1027/1016-9040.10.3.209

[B27] Karwowska D. (2007). O możliwościach modyfikowania i ograniczania wpływu nieuświadamianego afektu – rola refleksyjnego systemu przetwarzania, in Nieuświadomiony Afekt. Najnowsze Odkrycia, ed Ohme R. K. (Gdansk: GWP), 211–218.

[B28] Karwowska D. Kobylińska D. (2014). Exploring the effects of suboptimal affective priming: enhancement and minimization. Front. Psychol. 5:499. 10.3389/fpsyg.2014.00499 24904508 PMC4033072

[B29] Kobylińska D. (2007). Automatyczna Kontrola Nieświadomych Emocji. Warszawa: Wydawnictwa, UW.

[B30] Kobylińska D. Duszyñska K. (2013). Wpływ automatycznie aktywizowanej kontroli versus ekspresji emocji na poziom ich odczuwania, in W Stronę Podmiotowości. O Emocjach, Tożsamości, Dobrych Uczynkach i Pożytkach Płynących z Porannego Wstawania, eds Szuster A. Maison D. Karwowska D. (Sopot: Smak Słowa), 57–68.

[B31] Kobylińska D. Dyderska A. (2009). Utajony afekt a strategie regulacji, in Z Badań nad Pograniczem Intelektu i Osobowości, eds Ledzińska M. Zajenkowski M. (Warszawa: Wydawnictwo Instytutu Psychologii PAN), 97–117.

[B32] Kobylińska D. Karwowska D. (2014). Assimilation and contrast effects in suboptimal affective priming paradigm. Front. Psychol. 5:498. 10.3389/fpsyg.2014.00498 24904507 PMC4033262

[B33] Kolańczyk A. (2007). Samokontrola i wpływy bodźców afektywnych na ocenianie. Psychol. Społeczna. 2, 7–22. 25472608

[B34] Kolańczyk A. Pawłowska-Fusiara M. (2002). Automatic correction or controlled processing of affective priming. Pol. Psychol. Bull. 33, 35-44. 26278979

[B35] Koole S. L. (2009). The psychology of emotion regulation: an integrative review. Cogn. Emot. 23, 4–41. 10.1080/02699930802619031

[B36] Koole S. L. Coenen L. H. M. (2007). Implicit self and affect regulation: effects of action orientation and subliminal self priming in an affective priming task. Self Identity 6, 118–136. 10.1080/15298860601118835

[B37] Koole S. L. Jostmann N. B. (2004). Getting a grip on your feelings: effects of action orientation and external demands on intuitive affect regulation. J. Pers. Soc. Psychol. 87, 974–990. 10.1037/0022-3514.87.6.974 15598118

[B38] Koole S. L. Rothermund K. (2011). I feel better but i don't know why”: the psychology of implicit emotion regulation. Cogn. Emot. 25, 389–399. 10.1080/02699931.2010.550505 21432681

[B39] Koole S. L. Webb T. L. Sheeran P. L. (2015). Implicit emotion regulation: feeling better without knowing why. Curr. Opin. Psychol. 3, 6–10. 10.1016/j.copsyc.2014.12.027

[B40] Kuhl J. (1992). A theory of self-regulation: action versus state orientation, self-discrimination, and some applications. Appl. Psychol. Int. Rev. 41, 97–129. 10.1111/j.1464-0597.1992.tb00688.x

[B41] Mauss I. B. Bunge S. A. Gross J. J. (2008). Automatic emotion regulation: cultural and neuroscientific considerations, in Regulating Emotions: Culture, Social Necessity and Biological Inheritance, eds Ismer S. Jung S. Kronast S. van Scheve C. Vanderkerckhove M. (London: Blackwell Publishing), 39–60.

[B42] Mauss I. B. Evers C. Wilhelm F. H. Gross J. J. (2006). How to bite your tongue without blowing your top: implicit evaluation of emotion regulation predicts affective responding to anger provocation. Pers. Soc. Psychol. Bull. 32, 589–602. 10.1177/0146167205283841 16702153

[B43] Mauss I. B. Tamir M. (2014). Emotion goals: How their content, structure, and operation shape emotion regulation, in The Handbook of Emotion Regulation, 2nd Edn. ed Gross J. J. (New York, NY: Guilford Press), 361–375.

[B44] Mauss I. E. Cook C. L. Gross J. J. (2007). Automatic emotion regulation during anger provocation. J. Exp. Soc. Psychol. 43, 698–711. 10.1016/j.jesp.2006.07.003

[B45] Molden D. C. (2014). Understanding priming effects in social psychology. an overview and integration. Soc. Cogn. 32, 243–249. 10.1521/soco.2014.32.supp.243

[B46] Murphy S. T. Zajonc R. B. (1993). Affect, cognition and awareness: affective priming with optimal and suboptimal stimulus exposures. J. Pers. Soc. Psychol. 64, 723–739. 10.1037/0022-3514.64.5.723 8505704

[B47] Nosek B. A. Smyth F. L. Sriram N. Lindner N. M. Devos T. Ayala A.. (2009). National differences in gender-science stereotypes predict national sex differences in science and math achievement. Proc. Natl. Acad. Sci. U.S.A. 106, 10593–10597. 10.1073/pnas.0809921106 19549876 PMC2705538

[B48] Ohme R. (2005). Nieuświadomiony Afekt: Najnowsze Odkrycia. Gdańska: GWP.

[B49] Ohme R. K. Jarymowicz M. Reykowski J. (eds.). (2001). Automatyzmy W Procesach Przetwarzania Informacji. Warszawa: Wydawnictwo IPPAN i SWPS.

[B50] Rotteveel M. Gierholz A. Koch G. van Aalst C. Pinto Y. Matzke D.. (2015). On the automatic link between affect and tendencies to approach and avoid: Chen and Bargh (1999) revisited. Front. Psychol. 6:335. 10.3389/fpsyg.2015.00335 25883572 PMC4382967

[B51] Sheppes G. Gross J. J. (2012). Emotion regulation effectiveness: what works when, in Handbook of Psychology, Volume Five: Personality and Social Psychology, eds Tennen H. A. Suls J. M. (New York, NY: Wiley), 391–406.

[B52] Sheppes G. Scheibe S. Suri G. Radu P. Blechert J. Gross J. J. (2014). Emotion regulation choice: a conceptual framework and supporting evidence. J. Exp. Psychol. 143, 163–181. 10.1037/a0030831 23163767

[B53] Teige-Mocigemba S. Klauer K. C. (2013). On the controllability of evaluative-priming effects: some limits that are none. Cogn. Emot. 27, 623–657. 10.1080/02699931.2012.732041 23075050

[B54] Watson D. Clark L. A. Tellegen A. (1988). Development and validation of brief measures of positive and negative affect: the PANAS scales. J. Pers. Soc. Psychol. 54, 1063–1070. 10.1037/0022-3514.54.6.1063 3397865

[B55] Wentura D. Degner J. (2010). Automatic evaluation isn't that crude! moderation of masked affective priming by type of valence. Cogn. Emot. 24, 609–628. 10.1080/02699930902854587

[B56] Wentura D. Rothermund K. (2014). Priming is not priming is not priming. Soc. Cogn. 32 (Suppl), 47–67. 10.1521/soco.2014.32.supp.47

[B57] Williams L. E. Bargh J. A. Nocera C. C. Gray J. R. (2009). The unconscious regulation of emotion: nonconscious reappraisal goals modulate emotional reactivity. Emotion 9, 847–854 10.1037/a0017745 20001127 PMC2796590

[B58] Yong D. (2012). Replication studies: bad copy. Nature 485, 298–300. 10.1038/485298a 22596136

[B59] Zajonc R. B. (1980). Feeling and thinking: preferences need no inferences. Am. Psychol. 35, 151–175 10.1037/0003-066x.35.2.151

[B60] Zajonc R. B. (2004). An unmadiated phenomenon, in Feelings and Emotions, eds Manstead A. Frijda N. Fisher A. (Cambridge: Cambridge Univeristy Press), 194–203.

